# Control of neural probe shank flexibility by fluidic pressure in embedded microchannel using PDMS/PI hybrid substrate

**DOI:** 10.1371/journal.pone.0220258

**Published:** 2019-07-24

**Authors:** S. Rezaei, Y. Xu, S. W. Pang

**Affiliations:** 1 Department of Electrical Engineering, City University of Hong Kong, Hong Kong, China; 2 Center for Biosystems, Neuroscience, and Nanotechnology, City University of Hong Kong, Hong Kong, China; Arizona State University, UNITED STATES

## Abstract

Implantable neural probes are widely used to record and stimulate neural activities. These probes should be stiff enough for insertion. However, it should also be flexible to minimize tissue damage after insertion. Therefore, having dynamic control of the neural probe shank flexibility will be useful. For the first time, we have successfully fabricated flexible neural probes with embedded microfluidic channels for dynamic control of neural probe stiffness by controlling fluidic pressure in the channels. The present hybrid neural probes consisted of polydimethylsiloxane (PDMS) and polyimide (PI) layers could provide the required stiffness for insertion and flexibility during operation. The PDMS channels were fabricated by reversal imprint using a silicon mold and bonded to a PI layer to form the embedded channels in the neural probe. The probe shape was patterned using an oxygen plasma generated by an inductively coupled plasma etching system. The critical buckling force of PDMS/PI neural probes could be tuned from 0.25–1.25 mN depending on the applied fluidic pressure in the microchannels and these probes were successfully inserted into a 0.6% agarose gel that mimicked the stiffness of the brain tissue. Polymer-based neural probes are typically more flexible than conventional metal wire-based probes, and they could potentially provide less tissue damage after implantation.

## Introduction

Implantable neural probes are widely used to record and stimulate neural activities. These neural probes have been implanted into different tissues (e.g. brain, eye, and ear). Improving neural probe biocompatibility is one of the major tasks for many neural probe applications, especially for those that require long term usage. In recent years [1–5], biocompatible polymer-based neural probes have been showed to be promising for neural prosthetics. Different biocompatible polymers such as polydimethylsiloxane (PDMS) [6], parylene-C [7], liquid crystal polymer (LCP) [8], SU-8 [9–11], and polyimide (PI) [12–14] have been studied extensively as flexible neural probes to reduce human body responses after implantation [15–18]. These softer polymer-based neural probes tended to be difficult to be inserted into the targeted tissues [19]. For example, due to the high flexibility of PDMS, these neural probes were difficult to be inserted into the brain or some other targeted tissues, and they are used mostly for surface neural prostheses [6, 20, 21].

The flexibility of the neural probes is related to the degree of tissue damage induced during brain micro motions after the probes are implanted. Neural probes with higher flexibility could cause less tissue damage [18]. However, a minimum probe stiffness is needed to facilitate implantation into the brain tissue [22–24]. Previously, flexible probe insertion was accomplished using a probe stiffener, in which the flexible probe was attached to a rigid silicon (Si) substrate with a biodegradable glue such as polyethylene glycol (PEG) [25, 26]. Others had demonstrated that coating neural probes with bio-dissolvable materials [27] such as silk polymer [28, 29] could temporarily enhance neural probe stiffness during probe insertion.

Previous probe stiffening methods during insertion focused on using an extra piece of rigid structure to enhance the overall rigidity, rather than introducing dynamically controlled probe flexibility. In such cases, the neural probes cannot be reused after the bio-degradable layers were dissolved. Also, more tissue damage will be introduced due to the extra volume and stiffness of the rigid structure. Our present designs provide dynamic control of neural probe flexibility using adjustable fluidic pressure in the embedded channels. These neural probes were controlled to be rigid during insertion, and they returned to inherent flexibility after the insertion. The probes can be cleaned and reused easily. The neural probe flexibility was controlled by the probe materials, dimensions, and fluidic pressure in the embedded microchannels. In this work, neural probes were fabricated in PDMS, PI, and PDMS/PI hybrid substrates. The polymer-based neural probes are biocompatible and their mechanical properties can be optimized. The PDMS and PI polymers have different Young’s modulus, and they can provide different degrees of stiffness. Typically, PDMS is too soft for neural probe implantation into the brain tissue, while PI is more rigid compared to PDMS. A combination of PDMS and PI layers in neural probes could be tuned to reach the appropriate flexibility. For neural probe implantation, the fluidic pressure in the embedded microchannels of PDMS/PI neural probes could be adjusted to provide the needed stiffness. The fluidic pressure in the microchannels could then be released after implantation so that the neural probes will be highly flexible to reduce tissue damage. Probe flexibility for different designs as a function of fluidic pressure was simulated using the finite element method (FEM).

Neural probes in PDMS were too flexible to be implanted into brain tissue, even with fluidic pressure in the embedded channels. The minimum critical buckling force for the PI probes was found to be 3.0 mN for 200 μm wide probes with 50 μm wide channels. This is higher than the required force of 0.5–1.0 mN [30, 31] to penetrate into the brain tissue. However, hybrid PDMS/PI neural probes with the same dimensions and fluidic pressure were more flexible with lower critical buckling force of 1.1 mN, and rigid enough to penetrate into the brain tissue. The critical buckling force of the PDMS/PI neural probes could be tuned from 0.25–1.25 mN depending on the fluidic pressure in the microchannel. The fabricated probes were inserted into a 0.6% agarose gel that mimicked the softness of the brain tissue. As the fluidic pressure was increased from 0 to 60 kPa, these probes were able to penetrate into the gel. After insertion, releasing the fluidic pressure allowed the probe to become flexible so as to avoid tissue damage for chronic recording and stimulation.

## Materials and methods

In this section, the designs, materials and fabrication technology for PDMS/PI hybrid probe were discussed. Moreover, the flexibility in terms of axial critical buckling force of these neural probes was measured using the buckling test that resembled the probe insertion into the targeted brain tissues. A smaller critical buckling force indicated the probe was more flexible.

**Fig 1:**
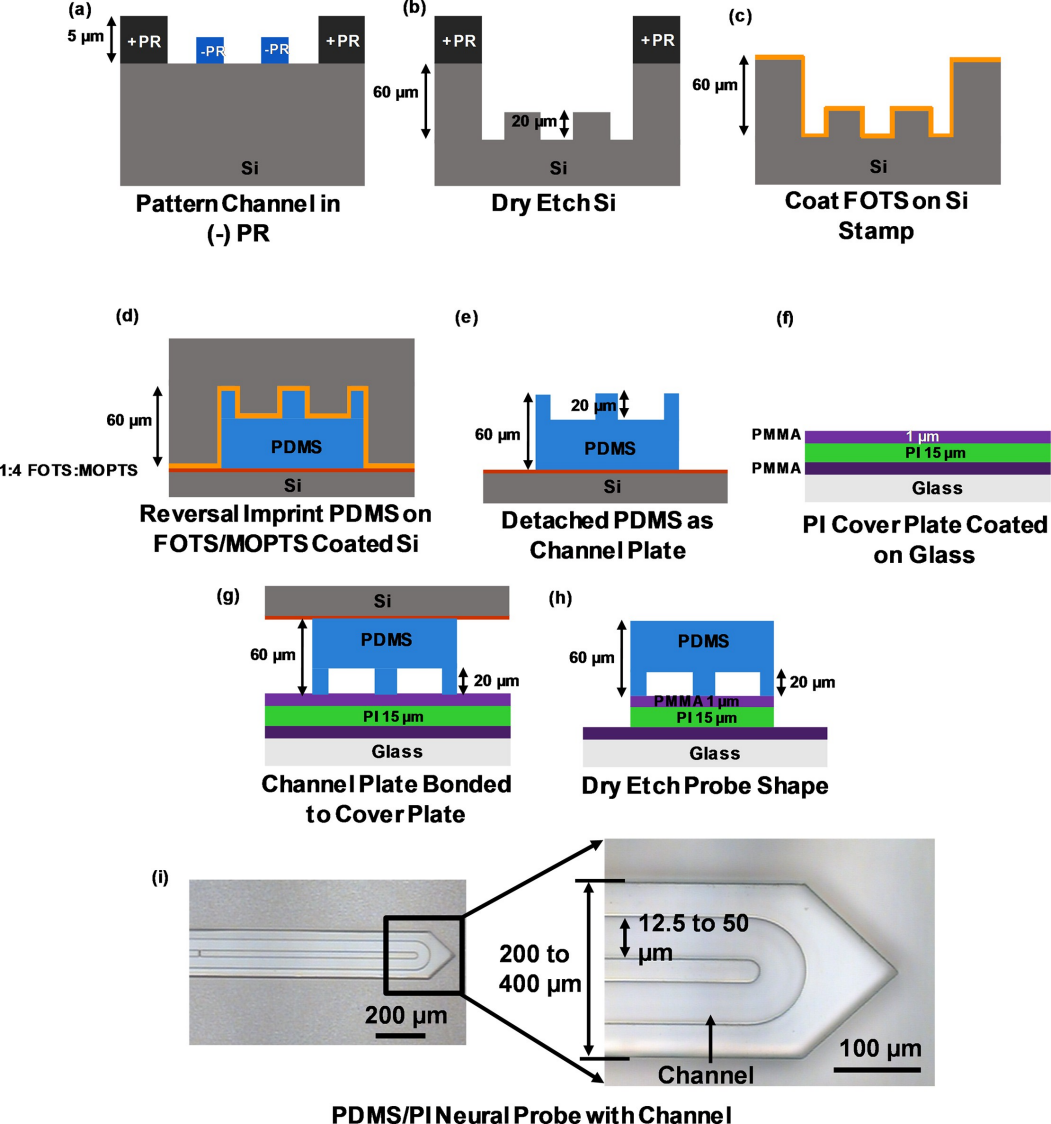
Fabrication of polydimethylsiloxane (PDMS)/polyimide (PI) neural probe with embedded microchannel. (a) Channels and probe shape patterned by 1 μm negative photoresist (-PR) and 5 μm positive photoresist (+PR), respectively, on silicon (Si). (b) 20/40 μm channel/probe shape dry etched in Si. (c) Photoresist was removed and trichloro (1H, 1H, 2H, 2H-perfluorooctyl) silane (FOTS) was coated on stamp. (d) and (e) 60 μm PDMS was coated on Si stamp and reversely imprinted on FOTS/methacryl oxypropylene trichlorosilane (MOPTS) coated Si and as channel plate. (f) 10 μm PI on poly(methyl methacrylate) (PMMA) coated glass with 1 μm PMMA coating as bonding layer. (g) PDMS channel plate was treated by O_2_ plasma and bonded to cover plate. (h) Probe shape was defined on cover plate by O_2_ plasma etching. (i) Released PDMS/PI neural probe with embedded microchannel.

The dimension of fluidic channel needed to be large enough to provide a large range of flexibility control, and bonding area needed to be large enough to ensure there was no channel leakage when pressure was applied. Thus, probe widths of 200, 300, and 400 μm was selected in this study. The embedded microchannels formed a ‘U’ shape in the neural probe as shown in Fig 1. With single channel width of 12.5, 25, and 50 μm on each side of the ‘U’, the total channel width was 25, 50, and 100 μm, respectively. The channel width and fluidic pressure in the channels determined the flexibility of the neural probes, which could be optimized for implantation and reducing tissue damage.

### Fabrication of PDMS/PI hybrid probes with embedded microchannel

The fabrication of the hybrid PDMS/PI neural probe is shown in Fig 1. The Si stamp with microchannel was fabricated for reversal imprinting PDMS channel plate and bonded on the PI cover plate using 1 μm thick poly(methyl methacrylate) (PMMA) (495PMMA A11, Micro-Chem) as an intermediate layer. For the channel shape in Si stamp, 1 μm thick AZ nLOF 2070 (diluted) negative photoresist was spin-coated on Si at 3000 rpm for 60 s and soft baked at 130°C for 60 s on a hot plate. The photoresist was patterned by UV exposure and post-baked at 130°C for 60 s. After the negative photoresist was developed, a 5 μm thick AZ 6130 positive photoresist was spin-coated at 3000 rpm for 60 s and baked at 90°C for 10 min. After patterning the positive photoresist, the Si stamp was ready to be dry etched (Fig 1A).

A deep reactive ion etching (DRIE) (LPX ICP LE0729, SPTS) system was used to etch the Si stamp in 2 steps. Initially, 20 μm Si was dry etched to define the channel depth and the remaining negative photoresist was removed using an O_2_ plasma. After the photoresist was removed, the Si stamp was etched for an additional 40 μm to realize 60 μm total channel plate thickness (Fig 1B). The remaining positive photoresist was removed in acetone for 10 min. The Si stamp was coated with trichloro(1H, 1H, 2H, 2H-perfluorooctyl) silane (FOTS) (Sigma-Aldrich, 97%), which acted as an anti-sticking layer [32] (Fig 1C). A 60 μm thick 10:1 (weight ratio, w/w prepolymer to curing agent) PDMS (Sylgard 184, Dow Corning) was spin-coated at 1000 rpm for 60 s on the Si stamp and reversely imprinted onto another Si substrate at 130°C and pressure of 40 bar for 10 min. The Si substrate surface was coated with 1:4 FOTS/ methacryl oxypropylene trichlorosilane (MOPTS) to provide higher surface energy than the FOTS coated Si stamp (Fig 1D). Due to different surface energy between the Si stamp and the Si substrate, the PDMS-based channel plate could be detached from the Si stamp (Fig 1E).

For the PI cover plate, 1 μm PMMA was spin-coated on the surface of a glass substrate at 3000 rpm and baked at 240°C for 30 min to facilitate the final peel-off of the neural probe from the glass substrate. Then 15 μm PI was spin-coated at 1000 rpm for 60 s and baked at 180°C for 5 h, followed by 240°C for 4 h. A 1 μm thick PMMA was spin-coated at 3000 rpm for 60 s as an intermediate bonding layer with the PDMS channel plate (Fig 1F). PDMS channel plate was treated by an O_2_ plasma (Plasma-Term 790 Series) using 25 sccm O_2_, 65 W stage power, and 700 mTorr chamber pressure for 20 s. The PDMS channel plate and the PI cover plate were bonded together with low pressure for 2 min, followed by baking at 130°C for 1 h (Fig 1G). An inductively coupled plasma (ICP) system (Oxford Instruments, Plasmalab 80 Plus) was used to dry etch 1/15 μm PMMA/PI at 10 sccm O_2_, 60 W stage power, 450 W Source power, and 10 mTorr chamber pressure for 15 min. PMMA and PI etch rate were 1.0 and 0.8 μm/min, respectively, with the PDMS layer as an etch mask. The entire PDMS/PI neural probe was detached from the glass substrate (Fig 1H). Fig 1I shows the neural probe with embedded microchannel after separation from the glass substrate. The width of the neural probes varied from 200 to 400 μm and channel width varied from 12.5 to 50 μm. 10 nm chromium adhesion layer and 200 nm gold layer were deposited on the PI followed by photolithography and wet etching to pattern the electrodes. The gold electrodes were located along the central line of the neural probe near the probe tip and they did not have overlap with the U-shaped channels. The gold electrodes did not have significant effect on the overall mechanical property.

### Probe flexibility: Critical buckling force simulations and measurements

**Fig 2:**
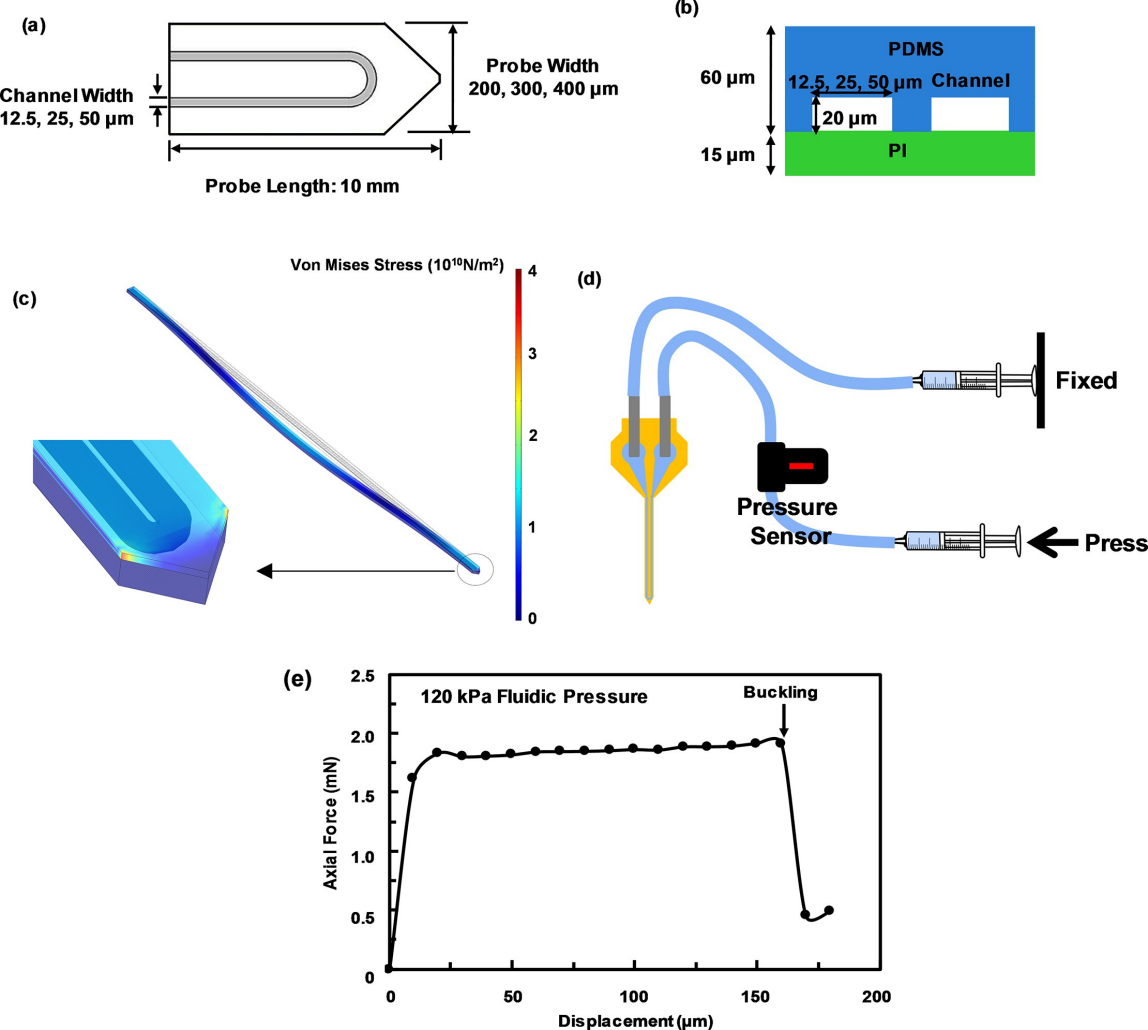
Stress distribution in neural probe based on finite element method simulation. (a) Probe geometry. (b) Probe dimensions. (c) Stress distribution on 200 μm wide, 1 cm long, 60/15 μm thick PDMS/PI probe shank with 50 μm wide, 20 μm high embedded channels during buckling. (d) Experimental setup of fluidic pressure control in U-shaped channel. (e) Measured axial force due to displacement of PDMS/PI neural probe with 400 μm probe width and 12.5 μm channel width at 120 kPa fluidic pressure.

Neural probes with different materials and dimensions were simulated using the FEM-based COMSOL MULTIPHYSICS 5.4 (COMSOL) [33]. Neural probes were modeled in 3-dimensional Cartesian coordinate system with 2-dimensional views shown in Fig 2A and 2B. The probe head was assumed to be fixed during the buckling. However, the probe tip was assumed to be sharp and could only move along the probe shank direction and could not slide along the surface. To study stress distribution around neural probe, PI and PDMS were set as the linear elastic and hyperelastic materials, respectively. PDMS followed the Neo-Hookean nearly incompressible material model with initial bulk modulus of 2.2 GPa [34]. Other material properties were directly imported from the material library. Simulation was performed in a sequence of two studies. In the first study, pressure ranging from 0–200 kPa was applied on the inner boundaries of the embedded channels to obtain the probe deformation and stress distribution. In the second study, a linear buckling analysis was performed where results from the first study were imported, and a force along the probe shank direction with a magnitude of 1 mN was applied on the tip of the probe. Critical buckling force was calculated by multiplying 1 mN with the exported critical load factor. The simulated von Mises stress distribution and the deflection of the PDMS/PI probe with 200 μm probe width and 50 μm channel width without pressure was shown in Fig 2C.

The critical buckling force was determined by measuring the force exerted by the neural probes on a microbalance (Sartorius BP 211D, Sartorius AG, Germany) with resolution of 0.01mg. At the initial contact between the probe and the balance, the force value was set to zero. Two syringes filled with water were connected to the inlet and outlet located at the two ends of the U-shaped channel via a pressure sensor. Fluidic pressure was applied by fixing the plunger of one syringe, while slowly pushing the other plunger until the required pressure was reached (Fig 2D). Fig 2E shows the changes of the axial force during the buckling test for a 60/15 μm thick PDMS/PI neural probe with probe width of 400 μm, channel width of 12.5 μm, and 120 kPa fluidic pressure in the microchannel. During the buckling test, after the probe tip contacted the microbalance surface, the probe shank gradually deformed as the probe was pushed down onto the microbalance by the induced force. At the threshold force of 1.9 mN, the probe buckled and the probe tip moved. All the critical buckling force was obtained based on the first buckling mode.

### Data analysis

All numerical data was shown in average value with standard deviation. Data was compared by Student's t-test, and two groups of data were considered to have significant difference when p <0.05 [35].

## Results and discussion

In this neural probe design, the U-shaped channel was filled with liquid. The total occupied space of the channel is the largest when there were no bending or other types of probe shank deformation. When the channel was bent under external force, this space would shrink accordingly. The fluidic pressure is an indicator of how well the channel is filled. A higher pressure indicated that the channel was filled with fluid that could stand against larger external force, thus more difficult for the probe to deform. In this case the overall stiffness of the probe became higher.

Table 1 shows the comparison of this work with other works in controlling neural probes flexibility. This work is the only one that provided dynamic control of probe flexibility using adjustable fluidic pressure in the microchannels. In addition, the neural probes in this work included PDMS and PI polymers, as well as various probe dimensions. Therefore, these probes could be optimized to the desirable low flexibility needed for implantation and high flexibility needed for *in vivo* operation with low tissue damage.

**Table 1:** Comparison of controlling neural probes flexibility in terms of critical buckling force.

Authors	Materials	Probe Size	Channel Size	Flexibility Control	Buckling Force	Insertion Force
This work	PDMS/PI	60 μm thick; 200–400 μm wide; 1 cm long	12.5–50 μm wide; 20 μm deep	PDMS/PI; fluidic pressure in channels	0.5–2.1 mN	0.8 mN at 60 kPa
Lecomte et al., 2015 [26]	Parylene-C	24 μm thick; 120 μm wide; 3 mm long	–	Biodegradable PEG and silk	2.6–300 mN	8–23 mN
Kim et al., 2014 [36]	Si/Parylene tube	100 μm thick; 120 μm wide; 3 mm long	–	Parylene tube dimension and location	540 mN	–
Jeon et al., 2014 [37]	Si	15 μm thick; 100 μm wide; 5.5 mm long	–	Breakable Si; biodegradable polymer	1.7 mN	–
Egert et al., 2011 [38]	Parylene	20 μm thick; 200 μm wide; 2 mm long	–	Parylene stiffener	0.5–1.3 mN	–
Takeuchi et al., 2005 [39]	Parylene	20 μm thick	50–200 μm wide; 10 μm deep	Biodegradable PEG in channel	12 mN	–

**Fig 3:**
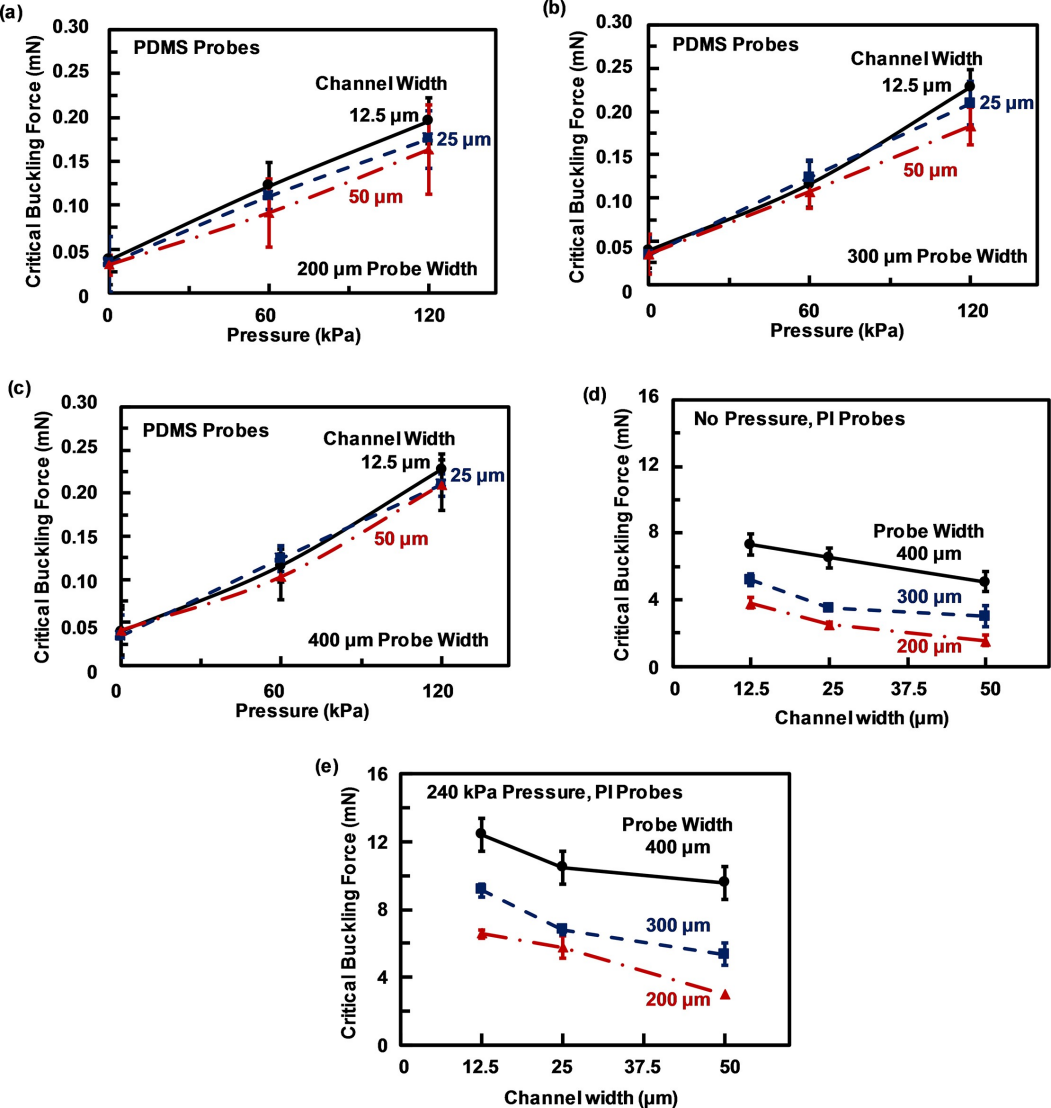
Critical buckling force of PDMS neural probes at different fluidic pressure. 60 μm thick PDMS neural probes with (a) 200, (b) 300, and (c) 400 μm probe width. Critical buckling force of 60 μm thick PI neural probes with channel width of 12.5, 25, and 50 μm; and probe width of 200, 300, and 400 μm with (d) no pressure and (e) at 240 kPa fluidic pressure. Fluidic pressure at 0 kPa means channel is filled with air while the inlet and outlet were not sealed.

Fig 3A to 3C show the critical buckling force for the PDMS-based neural probes with 200, 300, and 400 μm probe width, respectively. The critical buckling force for these PDMS-based probes were much smaller than the PI-based neural probes shown in Fig 3D and 3E. For 400 μm wide probes with 12.5 μm channel width, the critical buckling forces were found to be 0.06 and 7.30 mN for PDMS- and PI-based neural probes, respectively, with no fluidic pressure inside the channels. As channel width increased, the critical buckling force decreased for both types of probes. However, this effect was more significant for PI-based probes. The measurements also showed that the critical buckling force in the PDMS-based probes did not change significantly for probe widths varying from 200 to 400 μm due to PDMS’s hyperelastic properties. Further increase of the fluidic pressure to 240 kPa inside the embedded channel would cause channel deformation and swelling since PDMS was too flexible.

The critical buckling forces for 60 μm thick PI-based neural probes with different probe width and channel width are shown in Fig 3D and 3E without and with 240 kPa fluidic pressure. The critical buckling force for these PI-based neural probes decreased with decreasing probe width or increasing channel width. Fluidic pressure inside the microchannel could increase the critical buckling force significantly. For example, for 400 μm wide PI probe and 12.5 μm wide channel, the critical buckling force was 7.30 mN without fluidic pressure and it increased to 12.40 mN when 240 kPa fluidic pressure was applied. Similar increases were observed for 200 and 300 μm wide probes that the critical buckling force could be increased by applying fluidic pressure in the channels.

**Fig 4:**
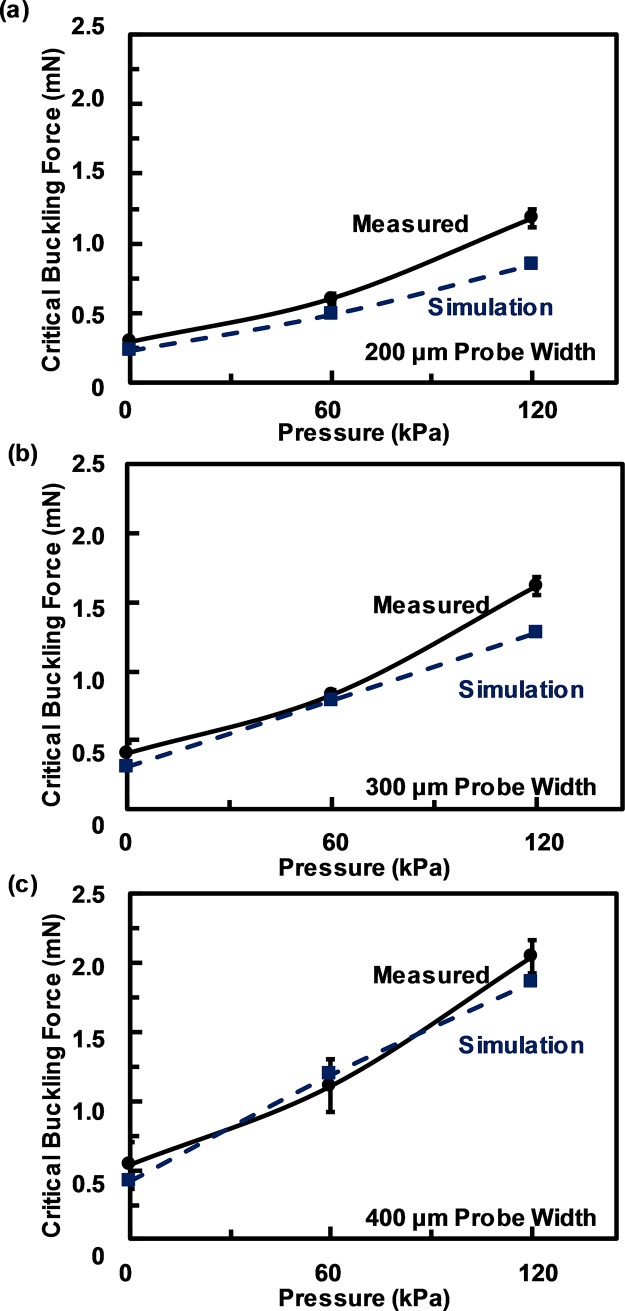
Measured and simulated critical buckling force of neural probes with channels at different fluidic pressure. Measured (solid line) and simulated (dash line) critical buckling force of 60/15 μm thick, 1cm long PDMS/PI neural probes with 12.5 μm wide embedded channels at different fluidic pressure for (a) 200, (b) 300, and (c) 400 μm wide probes.

Fig 4 shows the experimental and simulation results of the critical buckling force for the 60/15 μm PDMS/PI neural probe with 12.5 μm wide, 20 μm high channels. For 200 μm wide neural probe with 12.5 μm wide channel, the critical buckling force was 0.29 mN with no fluidic pressure and it increased to 0.60 mN when the pressure was increased to 60 kPa. As the pressure was increased further to 120 kPa, the critical buckling force continued to increase to 1.17 mN. The measured critical buckling force for neural probes at different pressures closely matched with the simulated results. Similarly, the measured and simulated results for 300 and 400 μm wide probes followed the same trends as shown in Fig 4B and 4C.

**Fig 5:**
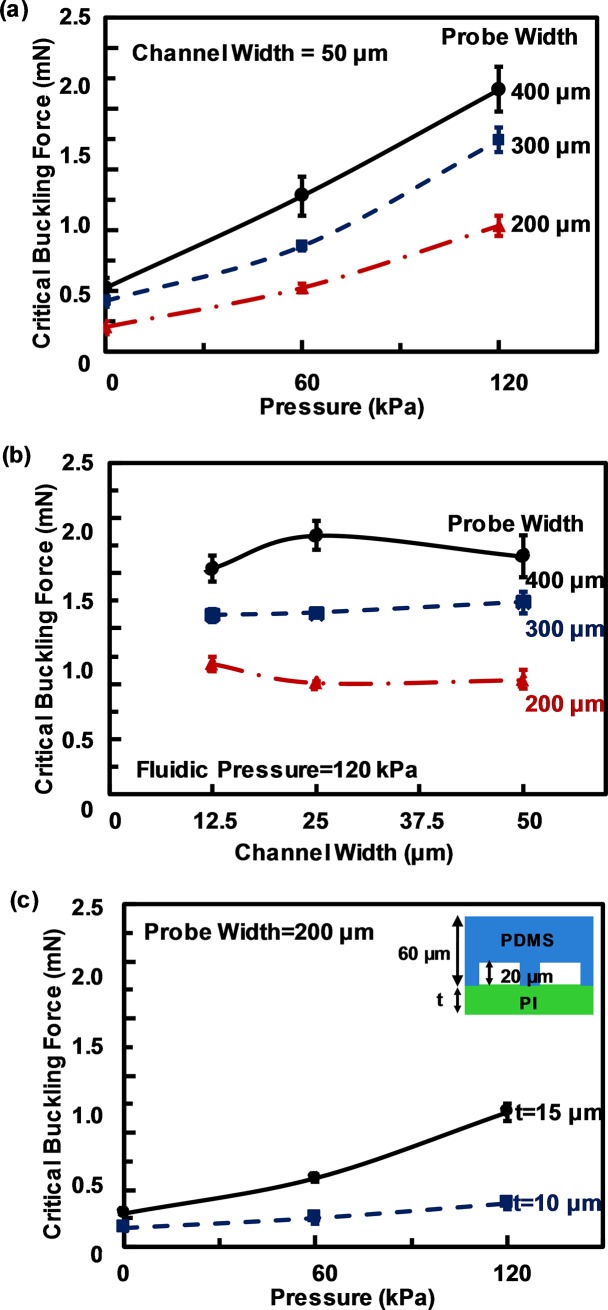
Measured critical buckling force of PDMS/PI neural probes. Measured critical buckling force of 60/15 μm PDMS/PI neural probes at (a) different fluidic pressure and (b) different channel width at 120 kPa. (c) Critical buckling force for PI thickness of 10 and 15 μm and probe width of 200 μm and channel width of 12.5 μm.

Fig 5A shows the effects of fluidic pressure on critical buckling force for neural probes with different channel widths and probe widths. The probes became stiffer with wider probes width, but the channel width had little effects on the critical buckling force as shown in Fig 5B. Channel width did not change the critical buckling force significantly because the probes consisted of 60 μm thick PDMS and only 10 or 15 μm thick PI layer with the channels. However, by changing the PI layer thickness from 10 to 15 μm the neural probes become stiffer as shown in Fig 5C.

**Fig 6:**
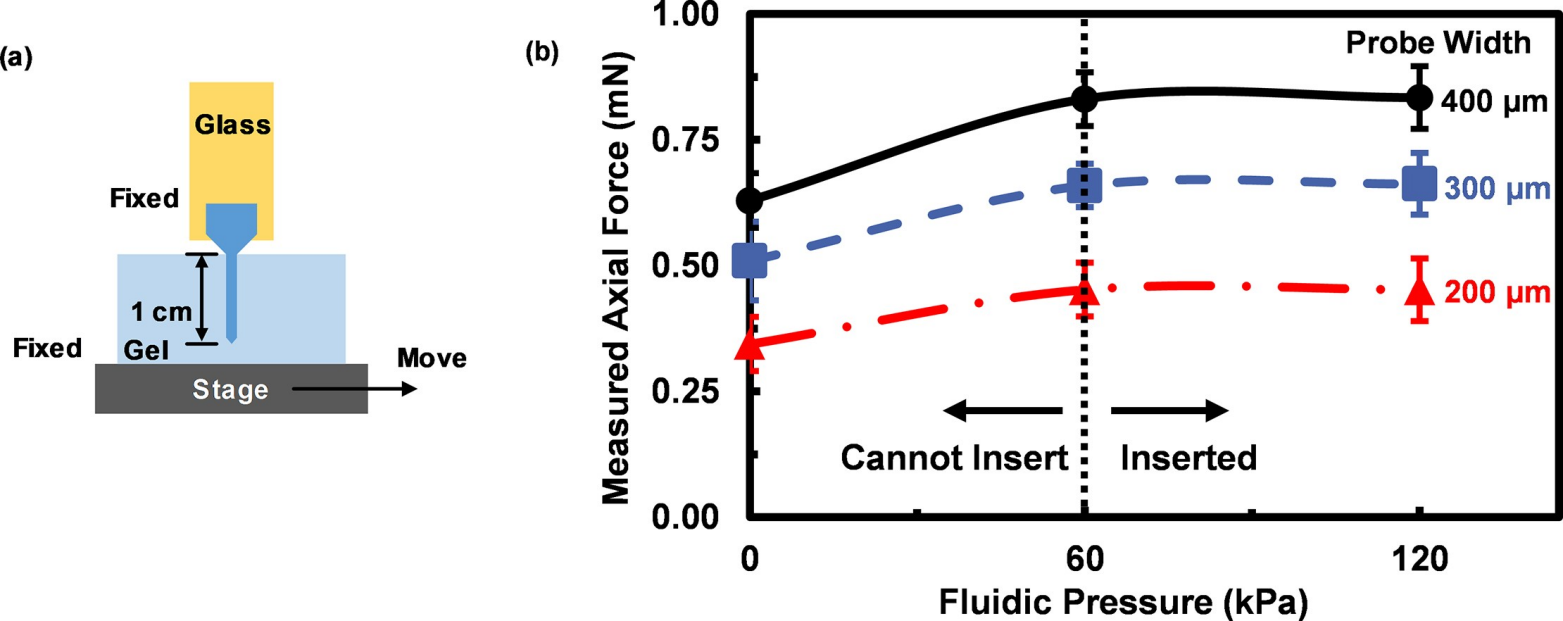
Measured axial force of neural probes in gel. (a) Schematic diagram showing gel movement setup. (b) Measured axial force during insertion in 0.6% agarose gel for 60/15 μm thick PDMS/PI neural probes at different fluidic pressure.

The fabricated probes were inserted into a 0.6% agarose gel that mimicked the brain tissue mechanical properties [40] as shown in Fig 6A. Measured axial force during insertion is shown in Fig 6B as a function of fluidic pressure in the channels. The neural probes could be inserted into the gel when the applied force was larger than 60 kPa to shear through the agarose gel. When no fluidic pressure was applied in the embedded microchannels, the probes were not able to penetrate into the gel. In this case the maximum applicable insertion force was found to be 0.34±0.05, 0.51±0.08, and 0.63±0.05 mN for 200, 300, and 400 μm probe width, respectively without bending the probe shank. With the small applied force, the gel surface was deformed by the probes without shearing through, and the probes were not able to penetrate into the gel. The penetration force for different probe design varies. Neural probes with smaller widths could be inserted more easily and required smaller insertion force compared to wider probes. Insertion force of the neural probes were in the range of 0.5–1 mN. Increasing the fluidic pressure to 60 kPa allowed the probes to shear through the gel surface with axial force of 0.45±0.06, 0.66±0.04, and 0.83±0.05 mN for 200, 300, and 400 μm probe width, respectively. Insertion force when further increasing the fluidic pressure to 120 kPa was 0.45±0.04, 0.66±0.06, and 0.83±0.06 mN for 200, 300, and 400 μm probe width, respectively, as shown in Fig 6B. No significant difference was shown between insertion forces under 60 and 120kPa pressure according to student’s t test. PDMS swelling was smaller than 20 μm with 120 kPa fluidic pressure for all designs. When pressure was higher than 120 kPa, more obvious PDMS swelling was observed, and leakage was found at PDMS/PI interface due to failure of bonding.

In order to characterize the flexibility of the neural probes, PI probes with width of 200, 300, and 400 μm were fabricated. Stiffness constant (*k*) in Nm^-1^ was calculated for all PI and PDMS/PI hybrid probes without applying any pressure by the following equations:

$$k=\frac{E_{c}wt^{3}}{4l^{2}}$$ (1)

$$E_{c}=\frac{E_{PDMS}V_{PDMS}+E_{PI}V_{PI}}{V_{Total}}$$ (2)

where *E*_*c*_ is the Young’s modulus of the PDMS-PI composite (Pa), *V*_*PDMS*_ and *V*_*PI*_ are the material volume, *w* is the probe width (m), t is thickness (m) and *l* is the probe length (m). The calculated stiffness constants were 1.68×10^−2^ to 3.37×10^−2^ Nm^-1^ for PDMS/PI probes and 0.17 to 0.35 Nm^-1^ for PI probes. The stiffness constant of PDMS/PI probes with the smallest dimension was comparable to the *k* value of 7.1×10^−3^ Nm^-1^ in the literature that showed significantly reduced tissue damage and glial formation [18]. Probes with larger width could potentially induce more damage to the nurons as well as more glial formation due to their larger area and higher rigidity. The flexibility performance of PDMS/PI probes can be further enhanced by optimizing the channel design, probe shape and size to provide better probe flexibility with smaller area to reduce tissue damage.

These probes were inserted into the gel and the gel was moved laterally, while the probe displacements were tracked using a camera. Nickel/chromium (Ni/Cr) and copper (Cu) metal wire probes with a diameter of 70 and 150 μm, respectively, were used to compare the probes flexibility. The micrographs of the probe positions inside the gel at their starting and ending positions after the gel was moved laterally by 4 mm were compared (S1 Fig). PI probes with width of 200, 300, and 400 μm had the displacement that decreased with increasing probe width, and the values were all close to 4 mm. Therefore the PI probes could move more easily with the induced motion and would not create as much tissue damage under motion. PDMS/PI probes were more flexible than the PI probes, thus the damage should be even smaller. In contrast, the displacement for the commonly used Ni/Cr and Cu wire probes were 3.21 and 0.35 mm, respectively. These results showed that the polymer-based neural probes were more flexible than the metal wire probes, which had the advantages of inducing less tissue damage due to brain micro-motions.

## Conclusions

This work demonstrated the utilization of embedded microchannels in neural probes to dynamically control their stiffness in order to reduce motion induced tissue damage. By adjusting the polymer materials, neural probe dimensions, and fluidic pressure in the microchannels, probe stiffness could be dynamically controlled so that they could be inserted easily into the targeted tissue, but returned to be flexible after implantation by changing the fluidic pressure. Therefore, using the combination of PDMS and PI layers in the developed neural probes with embedded microchannels, they have the advantages of controlling the probes to be stiff enough for insertion with pressure applied in the channels and becoming more flexible after penetration by releasing the pressure to potentially reduce tissue damage.

## Supporting information

S1 Fig Comparison of neural probe flexibility after insertion in agarose gel. (a) Displacement of neural probe tips after gel was moved for 4 mm. (b) Displacement for neural probe tips with 200, 300, and 400 μm wide PI probes and 70 μm nickel/chromium (Ni/Cr) and 150 μm diameter copper (Cu) metal wire probes.
(TIF)

S1 File Supplementary data showing simulated and measured results of neural probe flexibility. Flexible neural probes fabricated in PDMS/PI with microfluidic channels and electrodes. Critical buckling force of the probes was simulated and measured for various probe and channel dimensions, as well as different pressure in channel. Insertion force of neural probe in agarose gel and probe tip displacement were studied.
(PDF)
