# Supplementary figures and images for: Control of neural probe shank flexibility by fluidic pressure in embedded microchannel using PDMS/PI hybrid substrate
Source: PLoS One. 2019 Jul 24;14(7):e0220258. doi: 10.1371/journal.pone.0220258 (PMC6655783; doi:10.1371/journal.pone.0220258)

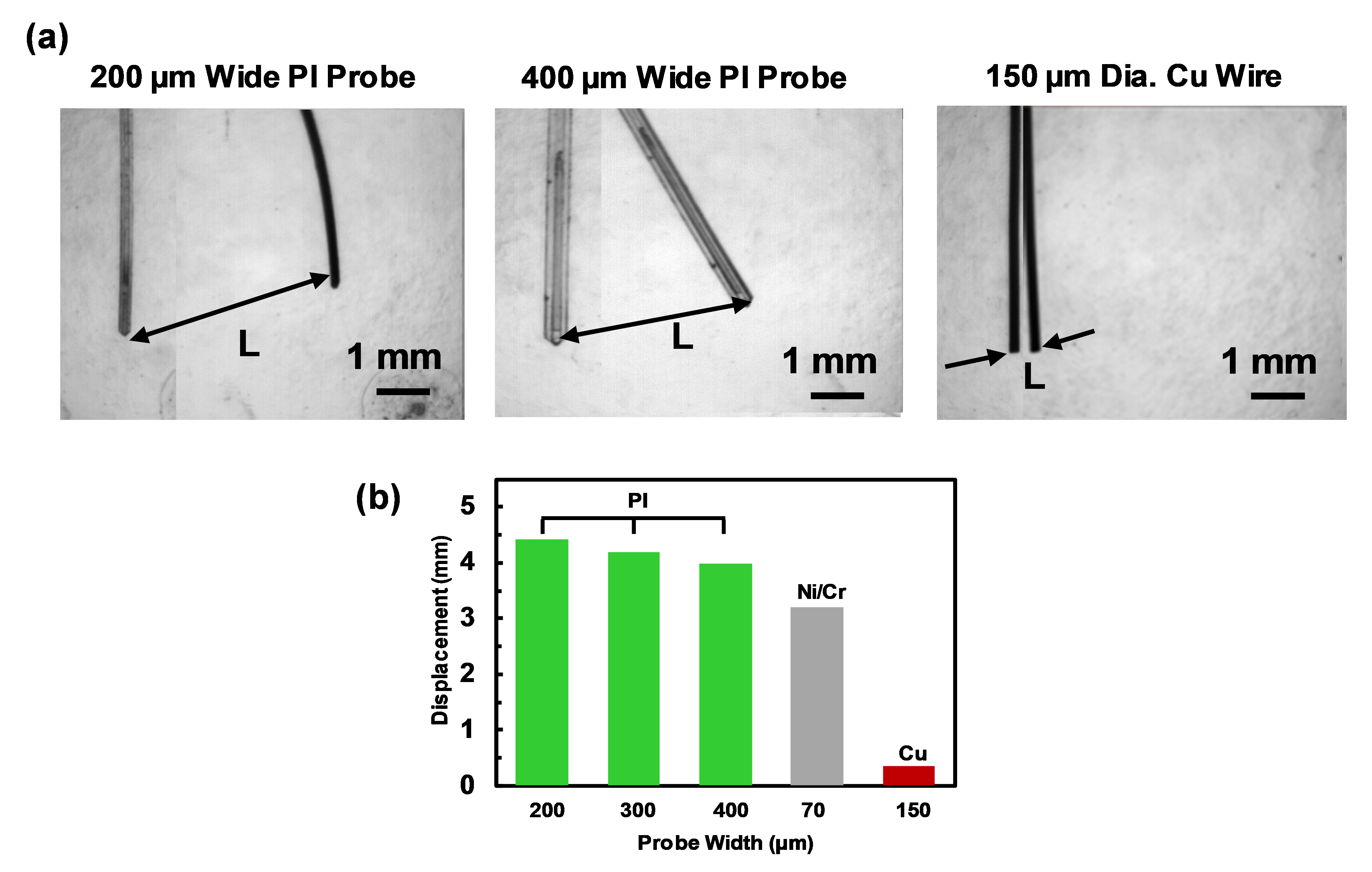

Supplement: S1 Fig — (a) Displacement of neural probe tips after gel was moved for 4 mm. (b) Displacement for neural probe tips with 200, 300, and 400 μm wide PI probes and 70 μm nickel/chromium (Ni/Cr) and 150 μm diameter copper (Cu) metal wire probes. (TIF) [file pone.0220258.s001.tif]
