# Supplementary material for: Control of neural probe shank flexibility by fluidic pressure in embedded microchannel using PDMS/PI hybrid substrate
Source: PLoS One. 2019 Jul 24;14(7):e0220258. doi: 10.1371/journal.pone.0220258 (PMC6655783; doi:10.1371/journal.pone.0220258)
Supplement: S1 File — Flexible neural probes fabricated in PDMS/PI with microfluidic channels and electrodes. Critical buckling force of the probes was simulated and measured for various probe and channel dimensions, as well as different pressure in channel. Insertion force of neural probe in agarose gel and probe tip displacement were studied. (PDF) [file pone.0220258.s002.pdf]

Control of neural probe shank flexibility by fluidic pressure in embedded microchannel using PDMS/PI hybrid substrate

S. Rezaei, Y. Xu, and S. W. Pang

Department of Electronic Engineering
Center for Biosystems, Neuroscience, and Nanotechnology
City University of Hong Kong, Hong Kong, China

PDMS/PI Neural Probe Fabrication

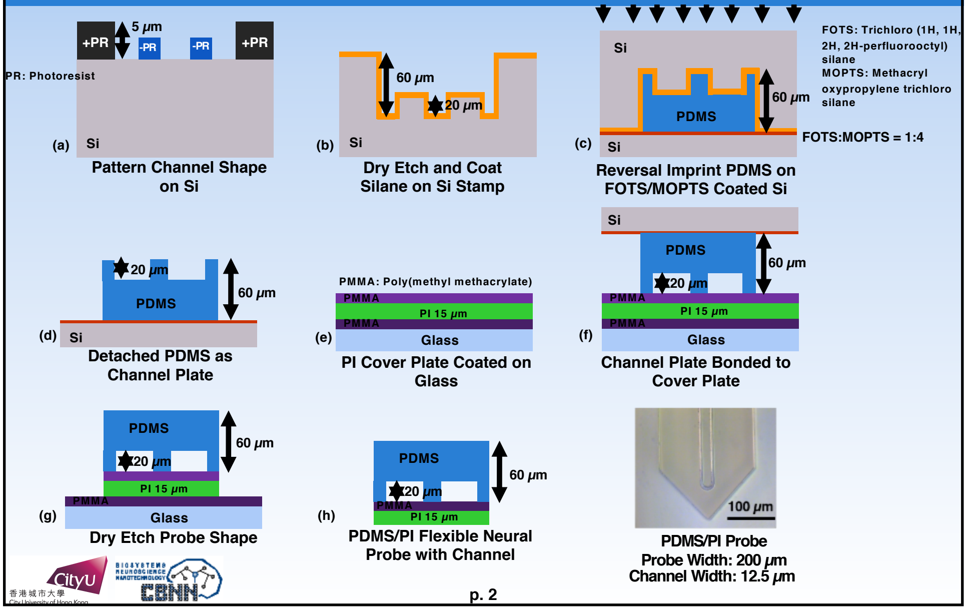

Neural Probes with Channels and Electrodes

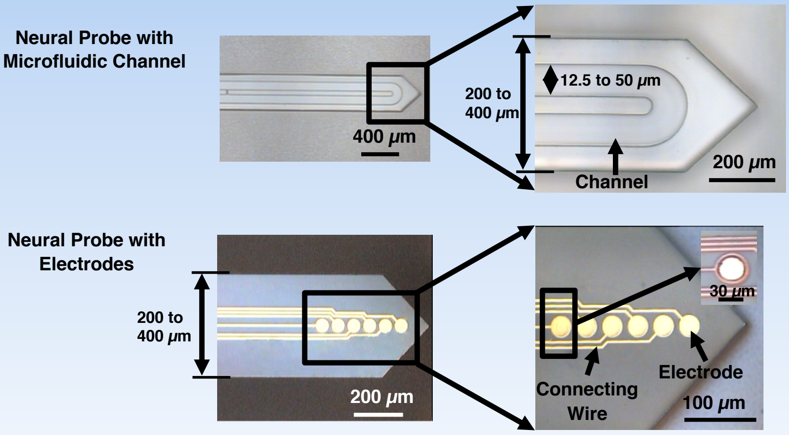

- Fluidic Channel was Fabricated in PDMS Using Reversal Imprint Technology
- Gold (Au) Electrodes were Fabricated in PI by Liftoff

Probe Critical Buckling Force Comparison between Simulation and Measurement

15/60 μm Thick PDMS/PI Probe, 20 μm Channel Depth, Probe Length: 10 mm
Probe Width: 300μm, Channel Width: 25μm

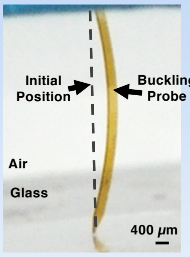

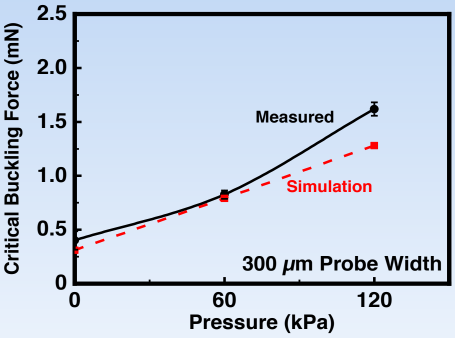

- Good Agreement Between Simulation and Measured Results

Fluidic Pressure Effect on PI Neural Probe Flexibility

60 μm Thick PI Probe, 1 cm Long, 25 μm Channel Depth

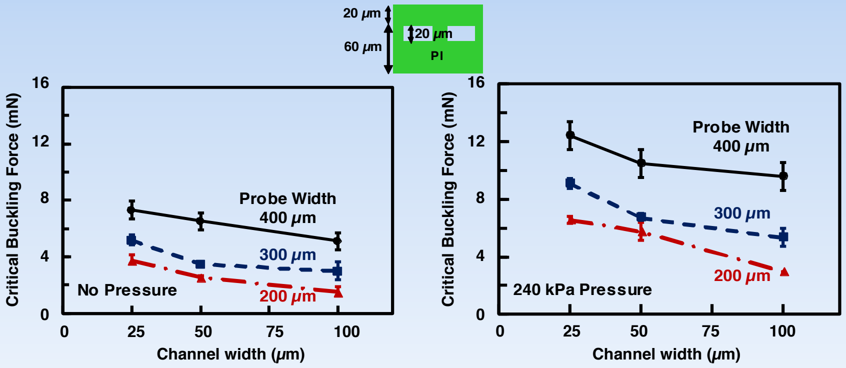

- Measured PI Probe Flexibility Decreased with Probe Width and Increased with Channel Width
- Probes Became Less Flexible with Fluidic Pressure in Channel

p. 5

PDMS Neural Probe Flexibility Adjustment by Fluidic Pressure

60 μm Thick PDMS Probe, 1 cm Long, 20 μm Channel Depth

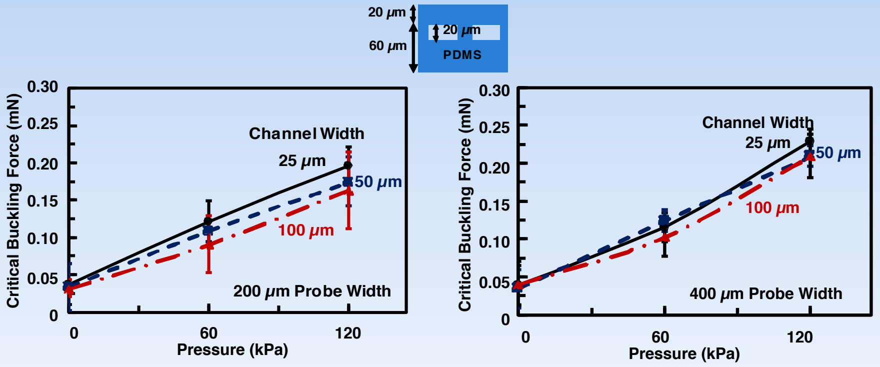

- Measured Probe Flexibility did not Vary with Channel Width for Soft PDMS Probes
- Probes were More Rigid at Higher Fluidic Pressure

p. 6

Dynamic Flexibility Control for PDMS/PI Neural Probes

15/60 μm Thick PDMS/PI Probe, 1 cm Long, 20 μm Channel Depth

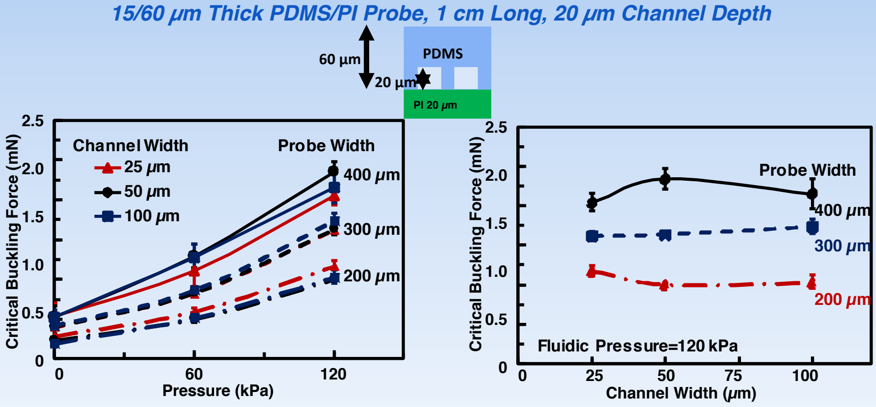

- Probe Flexibility did not Vary with Channel Width for Soft PDMS Channels
- Probes were Less Flexible at Higher Fluidic Pressure
- Decreased Probe Flexibility by Factor of 4 with 120 kPa Fluidic Pressure
- Decreased Probe Flexibility by Increasing Probe Width

CityU 香港城市大學 City University of Hong Kong
CBNN

p. 7

PI Cover Plate Thickness Effect on PDMS/PI Probe Flexibility Control

60 μm Thick PDMS, 1 cm Long, 20 μm Channel Depth, 25 μm Channel Width

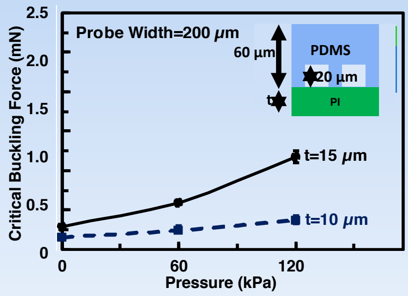

- Increased Probe Flexibility for Thinner PI Cover Plate
- Larger Range of Flexibility Control for PDMS/PI Neural Probes with Thicker PI Cover Plate

CityU 香港城市大學 City University of Hong Kong
CBNN

p. 8

Insertion Force for PDMS/PI Based Neural Probe

15/60 μm Thick PDMS/PI Probe, 20 μm Channel Depth, Probe Length: 10 mm
Probe Width: 200, 300, 400 μm; Channel Width: 100 μm, 0.6% Agarose Gel

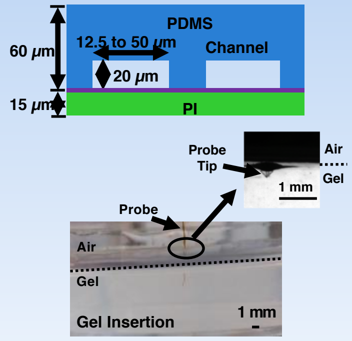

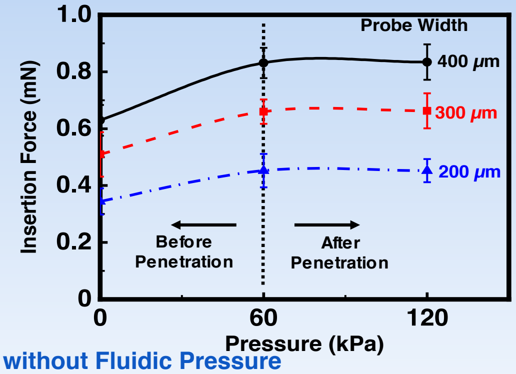

- Probes cannot be Inserted without Fluidic Pressure
- Probe Inserted Successfully when Fluidic Pressure was Applied in Embedded Channel
- Smaller Probe Width Required Lower Insertion Force

Probe Tip Displacement

60 μm Thick PI Probe, 25 μm Channel Depth, 10 mm Probe Length, 100 μm Channel Width
200 and 400 μm Wide PI Probe; 150 Diameter (Dia.) Copper (Cu)
0.6% Agarose Gel, Movement: 4 mm

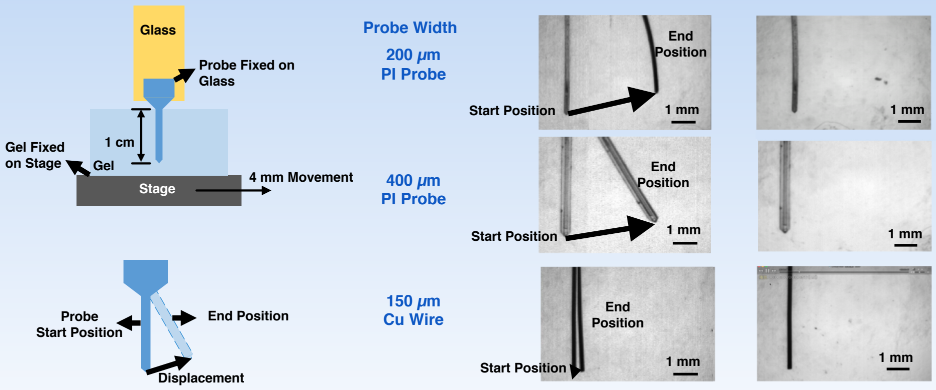

- Larger Displacement Means Probe could Move with Gel More Easily and have Better Flexibility

Neural Probe Flexibility Comparison

60 μm Thick PI Probe, 25 μm Channel Depth, 10 mm Probe Length, 100 μm Channel Width
200, 300, and 400 μm Wide PI Probe; 70 and 150 μm Dia. Au and Cu Wires
0.6% Agarose Gel, Movement: 4 mm

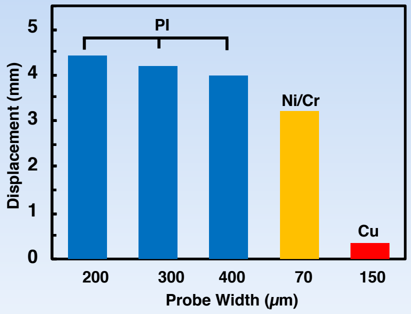

- PI Probes with Smaller Width were More Flexible
- Ni/Cr Wire with 70 μm Dia. was Less Flexible than PI Probes
- 150 μm Dia. Cu Wire was Less Flexible than 70 μm Dia. Au Wire

香港城市大學 City University of Hong Kong
